# Editorial: “Smart” rehabilitation technology and methodology to personalize neurorehabilitation

**DOI:** 10.3389/fresc.2023.1348086

**Published:** 2024-01-04

**Authors:** Ricardo Siu, James J. Abbas, Marika Demers, Carolee J. Winstein

**Affiliations:** ^1^ Department of Physical Medicine and Rehabilitation, Case Western Reserve University, Cleveland, OH, United States; ^2^ Institute for Integrative and Innovative Research and Department of Biomedical Engineering, University of Arkansas, Fayetteville, AR, United States; ^3^ Faculty of Medicine, School of Rehabilitation, University of Montreal, Montreal, QC, Canada; ^4^ Center for Interdisciplinary Research in Rehabilitation of Greater Montreal, Institut Universitaire en Réadaptation en Déficience Physique de Montréal-CIUSSS du Centre-sud de l’Ile de Montreal, Montreal, QC, Canada; ^5^ Division of Biokinesiology and Physical Therapy, Herman Ostrow School of Dentistry, University of Southern California, Los Angeles, CA, United States; ^6^ Department of Neurology, Keck School of Medicine, University of Southern California, Los Angeles, CA, United States

**Keywords:** neurorehabilitation, rehabilitation technology, closed-loop neuromodulation, personalized rehabilitation, precision medicine, individualized treatment, neurological injury

**Editorial on the Research Topic** “Smart” rehabilitation technology and methodology to personalize neurorehabilitation

Neurorehabilitation poses challenges for patients and providers. Whether triggered by a stroke, traumatic injury, or neurodegenerative disorder, the recovery journey is often prolonged and uncertain. Conventional methods, primarily one-size-fits-all, frequently fall short in addressing individual needs. The advent of “smart” rehabilitation technology marks a crucial shift towards personalized care, challenging the existing norms.

Recent research advances are enabling this transformative shift. Novel treatment paradigms, innovative medical algorithms, and an intensified focus on patient data are at the forefront. Our Research Topic compiles one perspective, one mini-review and five original research articles. These contributions explore tailored neuro-focused treatments, presenting new decision-making algorithms and investigating adaptive changes post-neural trauma. This compilation heralds a new era in neurorehabilitation, where patient needs are objectively met with precision, fostering more effective recovery.

The insightful review article by Bandres et al. highlights the significance of precision neuromodulation, specifically focusing on spinal stimulation for multi-modal rehabilitation. It stresses that while there have been advancements in spinal stimulation-based therapies for locomotor rehabilitation, there is a need to address the diverse sensorimotor deficits and priorities of individuals with spinal cord injury (SCI). The article concludes with addressing three potential areas that require attention for effective recovery: closed-loop neuromodulation to rehabilitate rather than replace, multi-modal rehabilitation paradigms for a more holistic and effective recovery, and the importance of considering the diverse patterns of intraspinal neural transmission inherent to each presentation. This highlights the broader relevance of personalized care in overcoming the limitations of current spinal stimulation therapies.

A mini-review by Wolpaw and Thompson guides us through how neurorehabilitation, with its focus on harnessing the plasticity of the central nervous system (CNS), holds great promise for personalized rehabilitation. This article emphasizes the importance of understanding skilled behaviors and the complex network of neurons and synapses involved in them, coined with the term “heksors”. By targeting beneficial plasticity in damaged heksors through noninvasive interventions, skill-specific practice can be enhanced, leading to wider beneficial plasticity and improved functional recovery. The persistence of these interventions' effects even after treatment ends highlights their potential for achieving more complete skill recovery and enabling personalized rehabilitation strategies.

A critical area in providing smart and personalized treatments for neurorehabilitation is the development of smart processes that are able to compute quantitative information to inform medical decisions rather than more traditional qualitative approaches. In this Research Topic, Swanson et al. found that exercise repetition rate measured with widely available commercial sensors can be used to estimate the Upper Extremity Fugl-Meyer (UEFM) score after a stroke. An exponential model was able to provide a good fit for estimating UEFM score based on repetition rate data collected in a clinical setting. This personalized approach has the potential to support self-directed home rehabilitation and reduce burden on clinicians by automating assessments.

Another subtype of algorithms that can adaptively control patient-specific functions when paired with methods such as functional electrical stimulation are also highly relevant to the concept of smart neurorehabilitation and are showcased in this Research Topic with two original research articles. Friedrick et al. present a study on stabilizing leaning postures in individuals with trunk paralysis using feedback-controlled functional neuromuscular stimulation (FNS). The personalized approach, incorporating muscle synergies and feedback control, allowed individuals to assume stable leaning postures to improve reaching capabilities and perceived stability. This research has significant implications for neurorehabilitation as it offers a personalized and effective strategy to enhance postural and functional abilities in individuals with spinal cord injuries. A similar approach, but geared towards restoration of respiratory function, was developed by Adury et al. They utilized an adaptive closed-loop neuromorphic controller to determine the timing and intensity of stimulation delivered to the diaphragm and external intercostal muscles. By simultaneously stimulating both sets of muscles, the study demonstrates reduced diaphragmatic fatigue and the ability of the controller to induce deep episodic breaths, i.e., sighs. This innovative strategy has the potential to meet a patient's specific respiratory needs, offering a promising alternative to traditional mechanical ventilation.

Lastly, original research by Wai et al. examined the changes in EMG activity patterns in Rhesus monkeys with cervical spinal cord injuries during various motor tasks. After the injury, there was an initial increase in activation levels and co-contraction of muscles, leading to poor coordination. However, over time, there was a reduction in EMG burst amplitudes and co-contraction, resulting in improved motor performance. These findings contribute to our understanding of motor recovery after spinal cord injury and may inform the timing of potential patient-specific therapeutic interventions.

The integration of “smart” rehabilitation technology in neurorehabilitation represents a journey that has potential to redefine healthcare. This paradigm shift merges cutting-edge technology with personalized, compassionate care. Celebrating our achievements, we must acknowledge the forthcoming challenges and ethical considerations. Data protection becomes paramount, demanding secure and confidential information gathering. Building patient trust hinges on stringent data protection measures and privacy standards, safeguarding individuals' interests. Accessibility stands as another looming challenge. Personalized neurorehabilitation should not be a privilege but accessible to all, transcending socio-economic and geographical boundaries. Bridging this digital divide necessitates collaboration among governments, healthcare institutions, and technology developers to democratize “smart” rehabilitation benefits for marginalized communities. Finally, it is critical to highlight that balancing technology advantages with the human touch is crucial. “Smart” rehabilitation technology should complement, not replace, healthcare providers' expertise and empathy. The human element, characterized by understanding, compassion, and adaptability to unique patient needs, must remain central in healthcare.

The future of neurorehabilitation is promising due to the incredible potential of “smart” rehabilitation technology. Embracing this innovation mandates a steadfast commitment to ethical responsibility, universal access, and preserving the human connection in healthcare. The synergy of technology and compassionate care will shape neurorehabilitation for years to come. Navigating the modern healthcare landscape involves more than improving treatment; it requires reimagining rehabilitation approaches. This transformation showcases human ingenuity's capacity to heal and enhance lives. “Smart” rehabilitation technology offers a future where recovery is personalized, inclusive, empowering, and transformative.

